# Topical Dosage Formulation of Lyophilized *Philadelphus coronarius* L. Leaf and Flower: Antimicrobial, Antioxidant and Anti-Inflammatory Assessment of the Plant

**DOI:** 10.3390/molecules27092652

**Published:** 2022-04-20

**Authors:** Ágota Pető, Dóra Kósa, Ádám Haimhoffer, Dániel Nemes, Pálma Fehér, Zoltán Ujhelyi, Miklós Vecsernyés, Judit Váradi, Ferenc Fenyvesi, Adina Frum, Felicia Gabriela Gligor, Laura Grațiela Vicaș, Eleonora Marian, Tunde Jurca, Annamaria Pallag, Mariana Eugenia Muresan, Zoltán Tóth, Ildikó Bácskay

**Affiliations:** 1 Department of Pharmaceutical Technology, Faculty of Pharmacy, University of Debrecen, Nagyerdei Körút 98, H-4032 Debrecen, Hungary; peto.agota@pharm.unideb.hu (Á.P.); kosa.dora@pharm.unideb.hu (D.K.); haimhoffer.adam@pharm.unideb.hu (Á.H.); nemes.daniel@pharm.unideb.hu (D.N.); feher.palma@pharm.unideb.hu (P.F.); ujhelyi.zoltan@pharm.unideb.hu (Z.U.); vecsernyes.miklos@pharm.unideb.hu (M.V.); varadi.judit@pharm.unideb.hu (J.V.); fenyvesi.ferenc@pharm.unideb.hu (F.F.); 2 Doctoral School of Pharmaceutical Sciences, University of Debrecen, Nagyerdei Körút 98, H-4032 Debrecen, Hungary; 3 Institute of Healthcare Industry, University of Debrecen, Nagyerdei Körút 98, H-4032 Debrecen, Hungary; 4 Faculty of Medicine, Lucian Blaga University Sibiu, Lucian Blaga Street, No 2A, H-550169 Sibiu, Romania; adinafrum@gmail.com (A.F.); feligligor@yahoo.fr (F.G.G.); 5 Department of Pharmacy, Faculty of Medicine and Pharmacy, University of Oradea, 29 Nicolae Jiga Street, H-410028 Oradea, Romania; laura.vicas@gmail.com (L.G.V.); marian_eleonora@yahoo.com (E.M.); jurcatunde@yahoo.com (T.J.); annamariapallag@gmail.com (A.P.); 6 Department of Preclinical Discipline, Faculty of Medicine and Pharmacy, University of Oradea, 1st December Square 10, H-410068 Oradea, Romania; marianamur2002@yahoo.com; 7 Department of Medical Microbiology, Faculty of Medicine, University of Debrecen, Nagyerdei Körút 98, H-4032 Debrecen, Hungary; toth.zoltan@med.unideb.hu

**Keywords:** *Philadelphus coronarius*, chemical compounds, penetration enhancers, cytotoxicity, ointments, topical application, antimicrobial, anti-inflammatory, antioxidant

## Abstract

*Philadelphus coronarius* is a versatile plant and its use in folk medicine has a long tradition; however, scientifically, the medical utilization of the herb is a less explored research field. The aim of our study was to identify and determine the quantity of the bioactive compounds of both the leaf and the flower and prepare a lyophilized product of them, from which medical ointments were formulated, since the topical application of *P. coronarius* has also not been studied. In vitro drug release, texture analysis and biocompatibility experiments were carried out, as well as the investigation of microbiological, antioxidant and anti-inflammatory properties. According to our results the composition and the selected excipients of the ointments have a great impact on the drug release, texture and bioavailability of the preparation. During the microbiological testing, the *P. coronarius* leaf was effective against *Escherichia coli* and *Staphylococcus aureus*, but it did not significantly decrease IL-4 production when it was tested on HaCaT cells. *P. coronarius* is a promising herb, and its topical application in antimicrobial therapy can be a useful addition to modern medical therapy.

## 1. Introduction

Herbs have an important role in human health because of their various beneficial therapeutic effects. Nowadays herbal compounds are becoming more and more favorable; however, natural does not necessarily mean better or safer. In order to use them effectively and safely, our current knowledge needs to be expanded [1,2].

*Philadelphus coronarius* (*P. coronarius*) belongs to Hydrangeaceae family, in the order of Cornales [3]. Many species of the Hydrangeaceae family are characterized by their antimicrobial and anti-inflammatory properties, but hepatoprotective and antidiabetic attributes have also been discovered [4,5,6]. *P. coronarius* is a less studied plant, but it is commonly used in folk medicine for a number of diseases, although the relevant scientific literature is very limited; only a few articles are available about its chemical elements and pharmacological characteristics. Previous chemical studies have revealed the flavonoid, triterpene, coumarin and phenolic-acid content of the herb [7]. *P. coronarius* is said to exhibit antibacterial effects, which is confirmed by the experience of folk medicine using it in the treatment of different gynecological illnesses [8]. The external application of the plant has never been investigated, even though it could be beneficial based on the above-mentioned facts, especially utilizing both the flower and the leaf.

In topical formulations, drug solubilization and sufficient penetration are critical factors. The proper pharmaceutical dosage form and the carefully selected excipients can support these parameters [9,10], and may modify drug penetration and bioavailability as well [11]. Excipients must be selected based on their ability to permeate into the skin, considering dermal toxicity and biological compatibility with the other components of the formulation [12,13].

**Figure 1:**
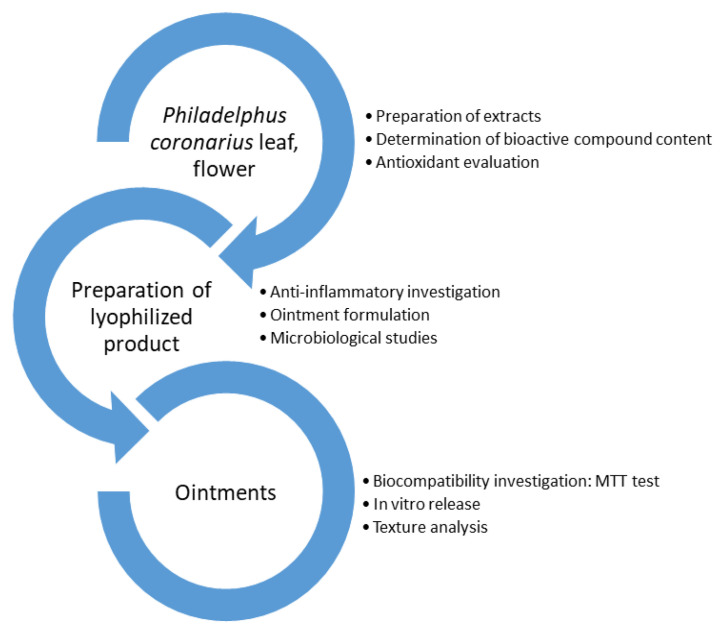
Experimental design.

The objective of our study (Figure 1) was to prepare lyophilized extracts from *P. coronarius* leaf and flower, to identify and standardize the bioactive components, as well as to evaluate the total antioxidant activity of the plant. Well-tolerable, six o/w emulsion-type ointments containing the lyophilized products were formulated with different emulsifiers. The selected penetration enhancers were incorporated into the preparations to ensure proper drug delivery and improve the bioavailability of the active pharmaceutical ingredient. Different dosage-form studies were performed with these formulations such as in vitro drug release, texture analysis, and biocompatibility experiments. Moreover, antimicrobial properties were investigated using the time-kill and microdilution methods. Anti-inflammatory properties of *P. coronarius* leaf and flower were studied with the help of enzyme-linked immunosorbent assay (ELISA). The main aim of our research was to develop a topical product for the market in the future, by taking advantage of the many beneficial effects of this herb. The novelty of this work is that we carefully characterized the active components of *P. coronarius* leaf and flower and certified their antioxidant and antimicrobial effects.

## 2. Results

### 2.1. Bioactive Content

As Table 1 presents, many phytochemicals were identified by the HPLC method. The leaf of *P. coronarius* contains a high amount of delphinidin 3-rutinoside chloride (0.3354 mg/100 mg), as well as luteolin 7-glucoside (0.2528 mg/100 mg) and 7-methoxycoumarin (0.2061 mg/100 mg) compared to the other components. Out of all the identified components, chlorogenic acid was detected in the smallest amount compared to the other compounds. 

Table 2 demonstrates the identified components of the *P. coronarius* flower. It contains bergapten in a high amount (2.8370 mg/100 mg), as well as caffeic acid (1.8407 mg/100 mg), delphinidin 3-rutinoside chloride (1.7928 mg/100 mg), 7-methoxycoumarin (1.6725 mg/100 mg). The flower contains delphinidin 3-rutinoside chloride and 7-methoxycoumarin in a much higher amount than the leaf. 

### 2.2. Antioxidant Activity of Philadelphus coronarius, Total Polyphenol and Flavonoid Content

Table 3 shows the antioxidant activity of the *P. coronarius* leaf and flower extracts evaluated with different methods. The flower exhibited significantly better antioxidant capacity with the ABTS (*p* ≤ 0.0241) and Cuprac methods (*p* ≤ 0.0093); however, with the DPPH and FRAP methods no significant differences were observed. The experiments were carried out in triplicate.

Table 4 represents the total polyphenol and flavonoid content of the flower and leaf extracts as the two main antioxidant components. The polyphenol and flavonoid content of the leaf and the flower was compared with a *t*-test, but the results did not significantly differ.

### 2.3. In Vitro Microdilution

Antimicrobial testing was carried out in order to study the antimicrobial potential of the *P. coronarius* leaf and flower by the microdilution method. Therefore, a lyophilized product of flower and leaf was dissolved in the relevant broth and the experiment was performed. As shown in Table 5, the flower was able to reduce the viability of *E. coli*, since cell viability was decreased to 46.4%. In the case of the leaf, it decreased cell viability of *S. aureus* to 68.6% and *E. coli* to 41.5%. Neither the *P. coronarius* flower nor the leaf inhibited the viability of *C. albicans* or *P. aeruginosa* in these experiments.

### 2.4. In Vitro Time-Kill Antimicrobial Test

**Figure 2:**
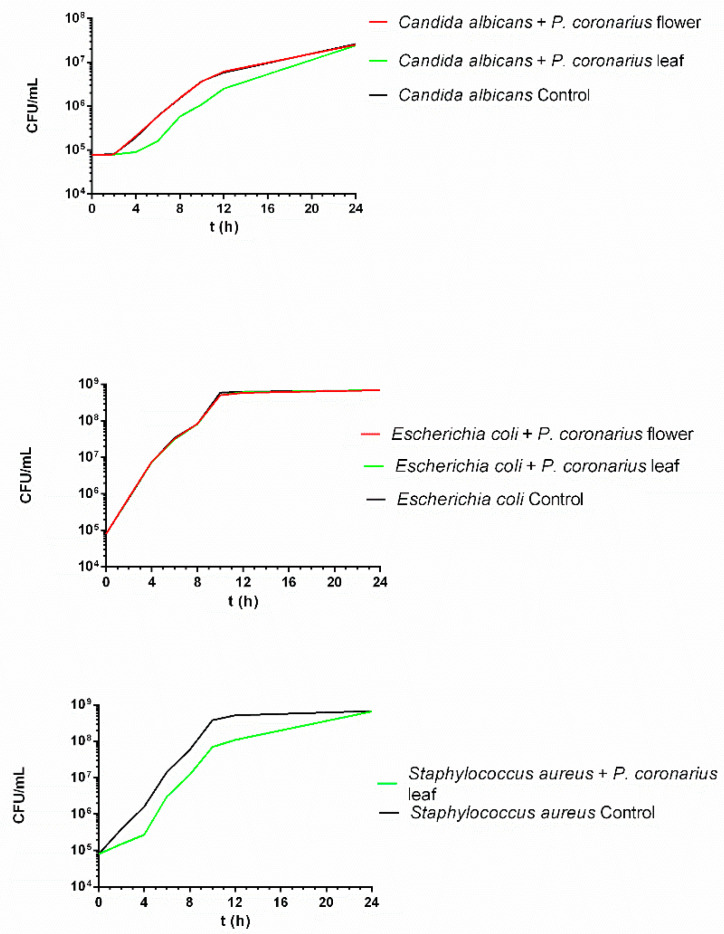
In vitro time-kill test of *P. coronarius* flower and leaf. The flower was not able to inhibit or delay the growth of bacteria or fungi, but the leaf was able to delay the growth of *C. albicans* and *S. aureus* compared to the control.

The time-kill test was performed to study the antimicrobial effect of the *P. coronarius* leaf and flower over time. Samples were prepared by dissolving the lyophilized product in the relevant broth (RPMI 1640 or Mueller-Hinton). At predetermined times, 100 μL of aliquots were plated on agar plates and counted. Killing activity was determined by the threshold of 99.9% (log_10_ CFU = 2.24) killing of initial CFUs. As Figure 2 demonstrates, *E. coli* was not affected by either the flower or the leaf, while only the latter was able to delay the growth of the *C. albicans* and *S. aureus* species. As a conclusion, none of the samples demonstrated any killing activity, and only a limited reduction in viable cell count was observed. 

### 2.5. Investigation of IL-4 Level, Enzyme-Linked Immunosorbent Assay (ELISA)

**Figure 3:**
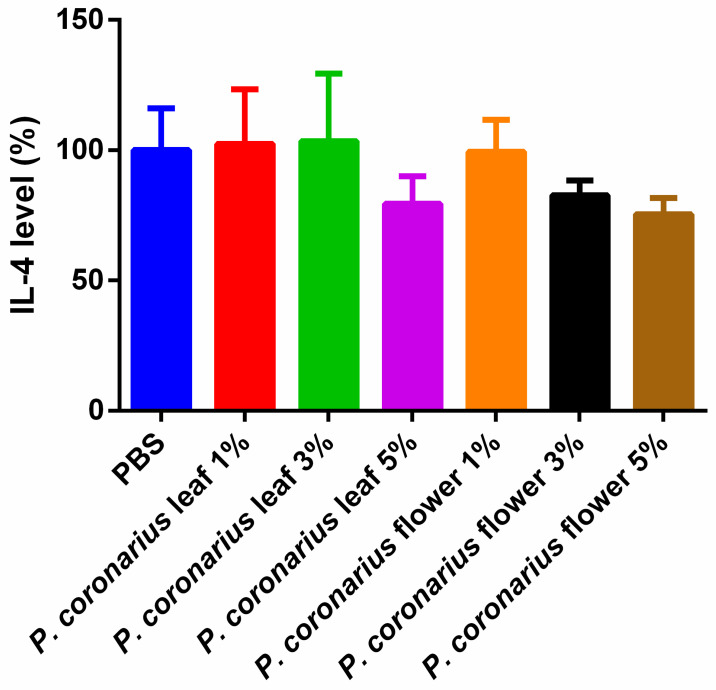
Results of IL-4 ELISA. Data represent the mean of six wells ± SD. Pretreating HaCaT cells with *P. coronarius* leaf (5%) and flower (3% and 5%) did not significantly reduce IL-4 production. Data are expressed as means ± SD; *n* = 5. To compare the groups, ordinary one-way ANOVA test was carried out.

The anti-inflammatory effect of the *P. coronarius* leaf or flower was studied using the ELISA test on HaCaT cells. Test solutions were prepared of lyophilized product dissolved in PBS (phosphate-buffered saline). During the study, PBS was selected as a negative control. It was taken as 100% during the evaluation and the values were compared and expressed as the percentage of the control. Pretreatment with the leaf (5%) and flower (3 and 5%) did not significantly reduce IL-4 production in the cells. Results are represented in Figure 3.

### 2.6. Ointment Formulation

O/w emulsion ointments were prepared containing the lyophilized extracts. Six compositions were formulated, with different emulsifiers and variations of lyophilized leaf or flower extracts (Table 6). The ointments were manufactured at the laboratory scale considering the final batch quantity and process parameters of the products.

### 2.7. In Vitro Release

**Figure 4:**
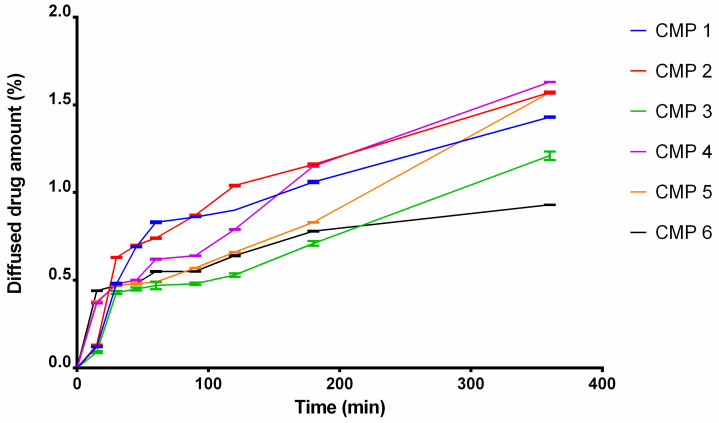
Release profiles of the different o/w ointment compositions. The best result was achieved by CMP 4, closely followed by CMP 2 and 5, while CMP 3 and 6 showed a greater sustained-diffusion profile in the experiment. Results represent the mean values of six samples ± SD.

Figure 4 demonstrates the diffused percentage of the active components of *P. coronarius* leaf and flower across a cellulose-acetate membrane. CMP 1, 2, 4 and 5 (compositions that contained Tefose 63 or SP70) showed the best results. The lowest release rate was achieved by CMP 3 and 6 (compositions, which contained Sedefos 75). Release rate (k) was determined from the slope of the amount of active substance released per unit area versus the square root of the time. Diffusion-coefficient values (D) were evaluated from the release-rate values. Kinetic model, release-rate values and diffusion-coefficient values are listed in Table 7 and Table 8. Release profiles of the preparations were compared to each other. Two formulations are different if their difference factor (*f1*) is between 0–15. Based on the calculated values, a difference is confirmed between CMP 1 and CMP 2. CMP 1 and 4, CMP 1 and 5, CMP 2 and 4, CMP 3 and 6, CMP 4 and 5, and CMP 5 and 6 are different as well. Difference factors are listed in Table 9.

### 2.8. Results of Texture-Analysis Studies

**Figure 5:**
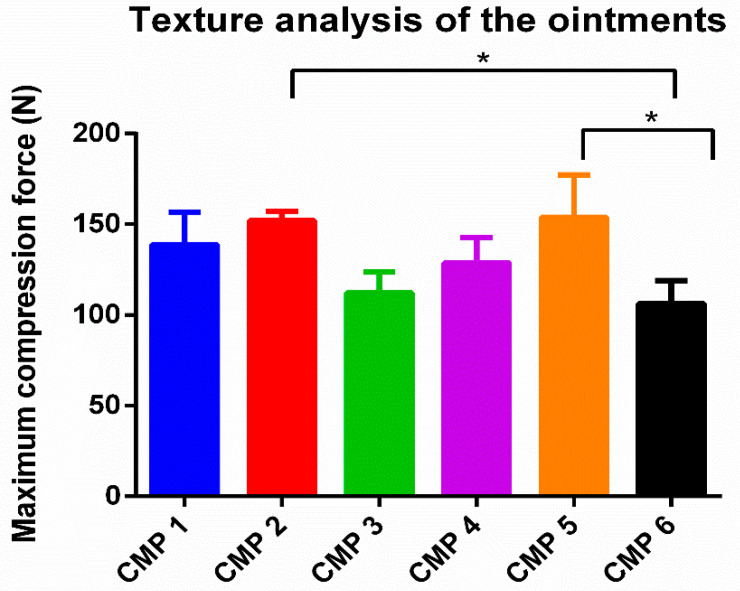
Results of texture-analysis studies. Resistance of the different ointments with the different surfactants. CMP 2 and 5 showed the hardest consistency, CMP 3 and 6 showed the softest consistency. Data are expressed as means ± SD; *n* = 5. To compare the preparations, ordinary one-way ANOVA test was carried out. Significant differences are marked in the figure with asterisks. * indicates significant difference at *p* < 0.05.

The maximum compression force needed for the cylinder to insert into the ointments is presented in Figure 5. Maximum compression-force values express the firmness of the preparations. Based on the results, CMP 2 and CMP 5 (which contain Tefose 63 as an emulsifier) had the hardest consistency (152; 153 N) compared to the other preparations. CMP 3 and 6 (which contain Sedefos 75 as an emulsifier) had the softest consistency of all (112, 106 N).

### 2.9. MTT Test

**Figure 6:**
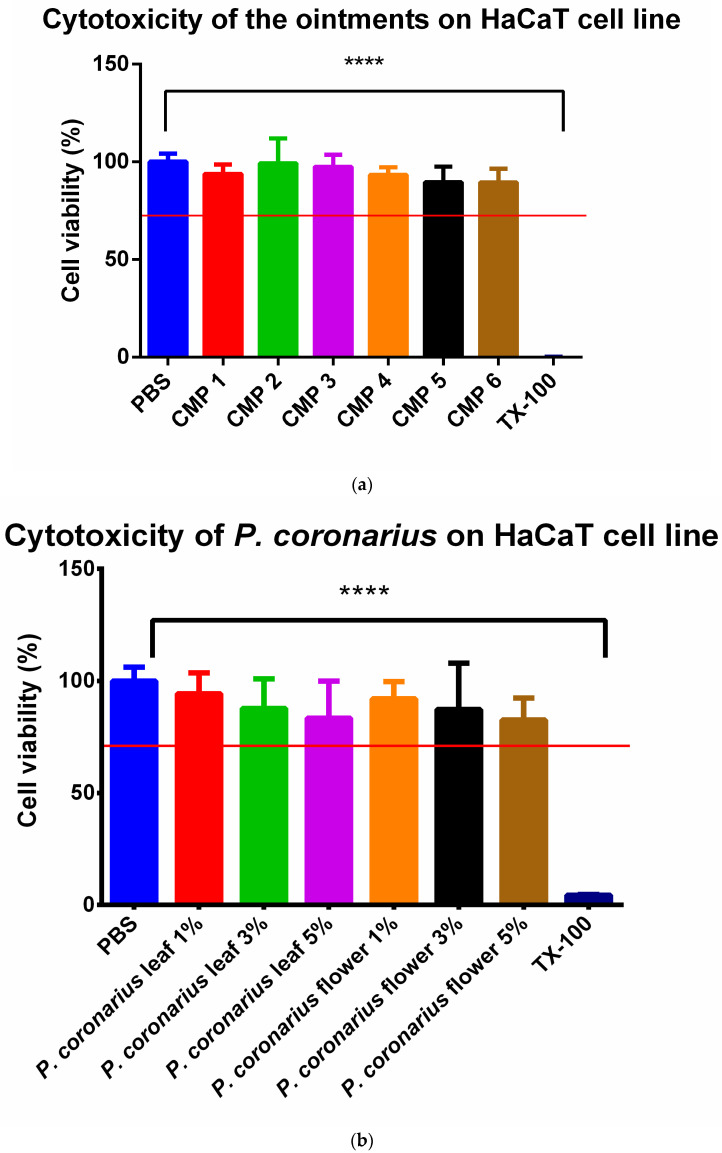
In vitro cytotoxicity of the prepared ointment compositions (**a**); in vitro cytotoxicity of *P. coronarius* leaf and flower in different concentrations (**b**). Cell viability was calculated as the percentage of PBS. All samples turned out to be safe; cell viability was above 80% in every case. Data present the mean of six wells ± SD. One-way ANOVA test and *t*-test were carried out for statistical analysis. Significant differences are marked with asterisks. **** indicates statistically significant difference at *p* ˂ 0.0001.

Results of the MTT test are presented in Figure 6a,b. The cytotoxicity investigation was performed separately for the formulated ointment compositions (a) and for the lyophilized products (b) on HaCaT cells. Cells were previously seeded on a 96-well plate. The experiment (a) was carried out with samples taken from the Franz diffusion-chamber apparatus, after 6 h of incubation, using pH = 5 buffer as the acceptor phase. For the other experiment (b), lyophilized product of either *P. coronarius* flower or leaf was dissolved in PBS at different concentrations. During both experiments, PBS was chosen as the negative control and Triton-X 100 as the positive control. Cell-viability values have been compared to the negative control (PBS) and expressed as the percentage of it. According to our results, CMP 2 produced the best results, but all test samples were safe and non-toxic, because cell-viability values were above 70% in each case (ISO 10993-5 recommendation). 

## 3. Discussion

In the present work, *Philadelphus coronarius* L. was characterized and investigated, because this plant was less studied in the literature. However, many beneficial pharmaceutical [14] effects such as anti-inflammatory [15] and antimicrobial activities were noticed during traditional applications [16]. As a novelty, both the leaf and the flower of the plant were utilized, and a lyophilized product was developed from the extracts. Additionally, antioxidant capacity was evaluated by different methods and a topical dosage form was formulated with the herb. 

In the case of natural preparations, well-characterized active-substance content, stability, and easy processability are crucial points. For these purposes, a lyophilized product was prepared. In this form, the active ingredients are stable and easy to process. The preparation of the lyophilized products is unique according to the scientific literature, since most studies have only investigated the extracts of *P. coronarius* [7,14,16], which still contains the organic solvent, e.g., ethanol and methanol, which may affect the safety and can lead to false conclusions. 

As part of our study, the antioxidant activity of *P. coronarius* leaf and flower was evaluated, as well as the total polyphenol and flavonoid content. Polyphenols are important metabolites and strong antioxidants that are able to protect against UV radiation and reduce inflammation [17,18]. Flavonoids act as antimicrobial agents and antioxidants, and they have anti-inflammatory effect as well [19,20]. The flower exhibited significantly better antioxidant capacity with the ABTS and Cuprac assays; however, no significant differences were observed with the DPPH and FRAP methods. Only the results of the ABTS and Cuprac assays were significantly different for the leaf and the flower, the main reason of which is presumably that more active components were detectable from the flower than from the leaf. Gallic acid and quercetin are two well-known, potent antioxidant compounds; they provide protection from oxidative stress [21,22,23,24]. These two compounds were only identified in the flower, which explains the better antioxidant capacity. Different antioxidant-activity-determining methods are based on different mechanisms and have varying sensitivities [25,26], which explains why no significant difference was detected between the antioxidant capacity of the leaf and flower by any method. This is why it is recommended to test antioxidant capacity by different methods.

According to the scientific literature, *P. coronarius* possesses antimicrobial properties. It has been previously studied by the microdilution method for Gram-positive and negative bacteria as well. It was confirmed that the extract had a strong antibacterial effect compared to the other herbal extracts in the study [16]. In our series of experiments, the antibacterial properties of the lyophilized flower and leaf were studied, followed by antifungal investigations. First, microdilution was carried out. In this investigation, the *P. coronarius* flower reduced the viability of *E. coli* and the leaf decreased the cell viability of *S. aureus* and *E. coli*. For further information in this topic, antimicrobial testing was continued with the in vitro time-kill test, whereby the effect of *P. coronarius* was studied on the growth of microbes over time. The growth of *S. aureus* and *C. albicans* was delayed by the leaf, while the flower did not elicit any changes. However, these results are not consistent with the results of the microdilution, since it is obvious that the leaf has antimicrobial potential. The time-kill test is considered to be the more accurate of the two methods, because it includes time as an important factor in the experiment.

The extracts from the *P. coronarius* leaf and flower were tested separately on previously inflamed HaCaT cells. The results show that premedication with the flower did not significantly reduce IL-4 levels, even though the anti-inflammatory effect of Hydrangeaceae species has been evaluated in the past. Dilshara et al. have investigated if *H. macrophylla* leaf extract had any anti-inflammatory effect [15]. It was observed that the extract suppressed the expression of some pro-inflammatory cytokines, e.g., NO, PGE_2_, and TNFα. Moreover, Nakamura et al. learned that *H. macrophylla* reduced the mRNS expression of IL-6 [6]. 

Carefully selected excipients may influence many properties of topical formulations, and in general they can also support the pharmaceutical effects by increasing the bioavailability [27,28]. When the ointment compositions were determined, excipients were chosen in order to achieve the best potential bioavailability of the active ingredients. According to Csizmazia et al., sucrose esters such as SP70 are able to enhance the penetration and the effect of an active substance, e.g., ibuprofen [29]. The same applies to Tefose 63; in the study of Abd-Elsalam et al., Tefose 63 improved the bioavailability and the effect of voriconazole [30]. It was also confirmed by our in vitro release studies. CMP 2 produced the best results, closely followed by CMP 1, 4, and 5. The lowest release-rate values belonged to CMP 3 and 6. According to these results, those formulations, which contained either Tefose 63 or SP70 as a surfactant, ended up having better release profiles, which may lead to better bioavailability of the active pharmaceutical ingredients. 

However, the application of surfactants and penetration enhancers may extremely influence the biocompatibility of different topical preparations. Cytocompatibility is a minimum requirement. It must be compulsory to check the safety of the preparation in each case. In our experiment, ISO-standard MTT tests were performed. Based on the results, the *P. coronarius* leaf and flower, as well as the ointment compositions were safe and non-toxic. The value of cell viability was over 70% in every case, which complies with ISO 10993-5 recommendations.

In the case of both pharmaceutical and cosmetic products, patient compliance is an important factor. The preparation must be as patient friendly as possible and little inconveniences must be reduced or avoided [31,32]. For ointments, texture may be part of the investigation of this compliance, therefore a texture analysis was performed. For this purpose, an o/w emulsion-type ointment was selected as the dosage form, since it is popular and preferred among patients due to its lighter consistency. The texture analysis revealed that CMP 2 and 5 had the hardest consistency (both contained Tefose 63), while CMP 3 and 6 was the softest (both contained Sedefos 75), but all compositions were easy to spread and apply.

## 4. Materials and Methods

### 4.1. Materials

SP70 sucrose ester was kindly gifted by Sisterna (Roosendaalc, The Netherlands). 3-(4,5-Dimethylthiazol-2-yl)-2,5-diphenyltetrazolium bromide (MTT paint), Dulbecco’s Modified Eagle’s Medium (DMEM), phosphate-buffered saline (PBS), Trypsin-EDTA, heat-inactivated fetal bovine serum (FBS), L-glutamine, non-essential amino-acid solution, and penicillin–streptomycin were purchased from Sigma Aldrich (Sigma Aldrich, St. Louis, MO, USA). Culturing flasks and 96-well plates were purchased from Corning (Corning, New York, NY, USA). Cetostearyl alcohol, propylene glycol, stearic acid, isopropyl myristate, and conserving solution were obtained from Hungaropharma Ltd. (Budapest, Hungary). HaCaT cells were supplied from Cell Lines Service (CLS, Heidelberg, Germany). Transcutol, Tefose 63, and Sedefos 75 were a kind gift from Gattefossé (Lyon, France).

### 4.2. Preparation of Lyophilized Products

Fresh leaves and flowers of *P. coronarius* were collected from the garden of University of Oradea, Faculty of Medicine and Pharmacy. A specimen of the *Philadelphus coronarius* L. species, young stem, leaves and flowers were kept in the Herbarium of the Faculty of Medicine and Pharmacy Oradea, Romania, registered in NYBG Steere Herbarium, UOP code 05313. 

With the purpose of obtaining the fluid extract, the maceration–extraction process at 20 °C was performed. The collected parts of the plant were dried in a dry room and protected from sunlight at room temperature. The product was fragmented and sieved with a No. III. pharmaceutical sieve. For the extraction, a mixture of ethanol and distilled water was chosen as a solvent (30 ^v^/_v_%). The mass ratio of the product to the solvent was 1:10 (^m^/_m_%). The extract was cleared from ethanol with the help of a rotary evaporator, Hei VAP Precision-Platinum 3, at 40 °C temperature, 80 rpm and 200 mBars. The remaining aqueous phase was frozen to −80 °C and then lyophilized (ALPHA 2-4 LSC plus lyophilizer) to gain a solid, water-soluble product [33].

### 4.3. Physicochemical Characterisation by HPLC-PDA Method

Identification and quantification of the bioactive compounds from *P. coronarius* extract were performed by using a Shimadzu Nexera-i LC–2040C 3D plus liquid-chromatograph system equipped with a photodiode array detector (PDA). A Phenomenex C18 (2) 100 A, 150 mm × 4.6 mm × 5 µm column was selected, and it was kept at 30 °C temperature. The mobile phases used for elution contained methanol (A) and formic acid 0.1% (B). The gradient program used was: 5% A and 95% B from 0 to 3 min, 25% A and 75% B from 3 to 6 min, 37% A and 63% B from 9 to 13 min, 54% A and 46% B from 18 to 22 min, 95% A and 5% B from 26 to 29 min and 5% A and 95% B from 30 to 36 min. The flow rate was 0.5 mL/min, and the injection volume was 10 µL. The detection was performed at multiple wavelengths: 254, 270, 275, 326, 337 and 360 nm. The polyphenols from the extract were identified by comparing the retention times from the extract chromatograms with the ones from the standard-solution chromatograms.

The bioactive-compound content was determined with the following equation:

(1) $$Bioactive\;compound\;content\;\left(mg/100mg\right)=\frac{m_{bioactive\;compound}}{m_{herbal}}$$

### 4.4. Phytochemical Investigation of the Lyophilized Extract

Bioactive-compound content of *P. coronarius* extracts (respectively, the total polyphenol and total flavonoid contents) were assessed. In order to calculate the total polyphenol content, the Folin–Ciocâlteu method was selected. It is based on the electron-transfer reaction, measuring the reductive capacity of an antioxidant. The outcomes of the Folin–Ciocâlteu method were very well correlated with the results attained from other antioxidant studies, such as ABTS and DPPH. Total polyphenol content was calculated as the gallic-acid equivalent (GAE/100 g) of dried plant based on the calibration line of gallic acid (5–500 mg/L, Y = 0.0027 × −0.0055, R^2^ = 0.9999). All determinations were performed in triplicate. For the assessment of total flavonoid content, the aluminum-chloride colorimetric method was selected. Quercetin was used for the standard calibration curve. The stock quercetin solution was made by dissolving 5 mg quercetin in 1 mL methanol, and afterwards a standard quercetin solution preparation with serial dilutions using methanol (5–200 µg/mL). Absorbance was measured at a wavelength of 420 nm with an UV–Vis Varia spectrophotometer. Total flavonoid concentration was calculated according to the calibration line (Y = 0.0162× + 0.0044, R^2^ = 0.999) and expressed as the quercetin equivalent (QE) mg/100 g in dried herb. All determinations were performed in triplicate.

### 4.5. Evaluation of Antioxidant Activity

#### 4.5.1. Evaluation of Antioxidant Activity by DPPH

The assay is based on the ability of the free radical DPPH to change its color in the presence of antioxidants. Antioxidant capacity of *P. coronarius* leaf and flower was tested. Samples were reacted with DPPH free radical in ethanol (96%). Reaction mixture contained 100 μL of *P. coronarius* leaf or flower test solution, 900 μL of ethanol and 2 mL of DPPH solution (0.06 mM). The reaction mixtures were incubated for half an hour. When DPPH reacts with an antioxidant, it changes its color from dark violet to yellow. Measurement of the remaining DPPH quantity was completed with UV spectrophotometer (Shimadzu Spectrophotometer, Tokyo, Japan) at the wavelength 517 nm. Purified water and absolute ethanol acted as background in 1:2 ratio. Scavenging activity was calculated according to Mensor et al. [34]:

(2) AA%=100−[((Abssample−Absblank)×100)/Abscontrol]

#### 4.5.2. Evaluation of Antioxidant Activity by FRAP Method (Ferric-Reducing Antioxidant Power)

FRAP method is a simple spectrophotometric technique that evaluates the antioxidant power of the test substance, being based on the reduction of ferric tripyridyltriazine complex [Fe(III)-TPTZ] by a reducer at an acidic pH. The stock solutions consisted of: 300 mM acetate buffer; 270 mg FeCl_3_·6 H_2_O dissolved in 50 mL distilled water; 150 mg TPTZ and 150 µL HCl, dissolved in 50 mL distilled water. FRAP solution was prepared by mixing 50 mL acetate buffer, 5 mL FeCl_3_·6 H_2_O solution and 5 mL TPTZ solution. Trolox was selected as a standard solution and absorbance was detected at 595 nm. The results are expressed as µmol Trolox equivalents (TE)/100 µL sample [35,36].

#### 4.5.3. Evaluation of Antioxidant Activity by CUPRAC Assay

We added 0.25 mL CuCl_2_ solution (0.01 M), 0.25 mL ethanolicneocuproine solution (7.5 × 10^−3^ M) and 0.25 mL CH_3_COONH_4_ buffer solution (1 M) and mixed the samples. Then, we adjusted the total volume to 2 mL with distilled water and mixed thoroughly, sealed the tubes, and kept them at room temperature. Absorbance was measured at 450 nm against a reagent blank 30 min later. Increased absorbance of the reaction mixture indicated increased reduction capability [37]. 

#### 4.5.4. Evaluation of Antioxidant Activity by ABTS Assay

ABTS (2,2’-azinobis-(3-ethylbenzothiazoline-6-sulfonic acid) was used to determine antioxidant activity of different compounds. ABTS assay is able to measure the ability of antioxidants to scavenge ABTS, compared to Trolox standard. ABTS was dissolved in water at a 7 mM concentration. ABTS radical cation was produced by reacting the stock solution of ABTS with potassium persulfate and letting the mixture stand in a dark room overnight. ABTS^+^ and the samples were diluted with ethanol. A total of 10 μL of diluted sample (or Trolox standard) was added to 1 mL of ABTS^+^. Absorbance values were measured with a spectrophotometer (Fluostar Optima) at a wavelength of 734 nm [38].

### 4.6. Antimicrobial Testing by Microdilution Method

Antifungal- and antimicrobial-tendency testing was carried out by using microdilution method against *C. albicans*, *P. aeruginosa*, *E. coli* and *S. aureus*. The experiment was performed by using 96-well standard microtiter plates and the concentrations of the tested compounds were prepared in RPMI-1640 and Mueller–Hinton medium for fungal species and bacteria. All test samples were prepared 10 min before starting the incubation. The final volume of each well contained 100 μL of the test compound and 100 μL of the fungal or bacterial inoculum. Plates were incubated for 24 h at 37 °C. After incubation, absorbance was measured at 492 nm and 600 nm. Inhibition was determined based on turbidity, in terms of at least a 50% growth reduction compared with the test-compound-free control. Percent change in turbidity was calculated by the following equation, where absorbance (A) was taken as 100% and the background was measured from the microbe free well [39]:

(3) $$A=\frac{\left(A\;\mathrm{well}-A\;\mathrm{background}\right)}{\left(A\;\mathrm{compound}\;\mathrm{free}\;\mathrm{well}-A\;\mathrm{background}\right)}\times 100$$

### 4.7. In Vitro Time-Kill Antimicrobial Tests

During antimicrobial testing, *E. coli*, *S. aureus* and *C. albicans* were chosen as reference strains. Activity of *P. coronarius* leaves and flower (dissolved in the relevant broth) was determined against *C. albicans* and bacteria strains in RPMI-1640 and Mueller–Hinton broth at 5 ^w^/_w_% concentration with a starting inoculum of 10^5^ cells/mL, in a final volume of 5 mL. In the case of *C. albicans*, 100 μL aliquots were removed at 0, 4, 8, 12, 24 h of incubation, ten-fold dilution series were prepared, and sample dilutions (4 × 30 μL) were plated onto Sabouraud dextrose agar plate and incubated for 48 h at 35 °C. In the case of *E. coli* and *S. aureus*, 100 μL aliquots were removed at 0, 2, 4, 6, 8, 10, 12, 24 h of incubation, ten-fold dilution series were prepared, and sample dilutions (4 × 30 μL) were plated onto Mueller–Hinton plates and incubated for 48 h at 35 °C [40].

### 4.8. Investigation of IL-4, Enzyme-Linked Immunosorbent Assay (ELISA)

To investigate if *P. coronarius* flower or leaves have any effect on inflammation, ELISA was performed on HaCaT cells. Cells were seeded on 96-well plates in the density of 10.000 cells/well. When the cells fully grow over the wells’ membrane, the experiment is ready to perform. Culture media was removed, then the cells were incubated with the samples for 1 h. Samples were made of lyophilized *P. coronarius* leaves or blooms dissolved in PBS, then filtrated (0.2 μm). Samples were prepared in different concentrations: 1 ^w^/_w_%, 3 ^w^/_w_%, 5 ^w^/_w_%. After samples were removed, 50 μL of TNFα (20 ng/mL) and 50 μL of IL-1β (25 ng/mL) were added to the cells and incubated with them over night. The next day the supernatant was removed, and a human IL-4 ELISA kit was performed according to the manufacturer’s instructions [41,42].

### 4.9. Formulation of Ointments

Different emulsifiers were incorporated into the formulations: sucrose esters (SP70) Tefose 63, Sedefos 75. The ointments were produced by melting stearic acid, cetostearyl alcohol, isopropyl myristate and mixed to prepare the oily phase of the formulation. The aqueous phase contained propylene glycol, emulgent, glycerol, and purified water, and was heated to the same temperature as the oil phase (~60 °C), mixed together and cooled down to room temperature. After that, lyophilized plant extract and conserving solution was added to the preparation. Lyophilized plant extracts were previously dissolved in a 1:1 mixture of transcutol and purified water [43]. The composition of the ointments can be found in Table 10.

### 4.10. In Vitro Release

In vitro release test was performed with the help of a Franz diffusion-chamber apparatus. During the test, a membrane is placed between the donor and the acceptor phase. The concentration profile of the test substance is obtained by taking samples at predetermined times. Samples weighing 300 mg were placed on artificial cellulose-acetate membrane (0.5 µm pore size) as the donor phase, and as the receptor phase, pH = 5 buffer was chosen in order to imitate the pH of the skin. The membrane was pretreated with isopropyl myristate to characterize the lipophilic property of the skin. The rotation of the magnetic stirrer was 450 rpm. To imitate the temperature of the skin, the receptor phase was held at 32 °C. *P. coronarius* content was measured with spectrophotometry [44,45]. Release rate (k) was determined from the slope of the lines fitted to the curves. Diffusion coefficient (D) was calculated from the amount of drug released per unit area (Q; μg/cm^2^), the initial drug concentration (C^′^_0_), and diffusion time (t) (Equation (4)):

(4) $$D=\frac{Q^{2\;}\times \pi }{{\left(2\left[C_{0}^{\prime }\right]\right)}^{2}\times t}$$

Data were fitted to zero-order and first-order kinetics (Table 7). Mathematical model of in vitro release is described in Table 11.

**Table 11:** Mathematical model of drug-release profiles.

Model	Equations [46,47]		Graphic
Zero-order	$Q_{t}=Q_{0}+k_{0}t$	(5)	The graphic of the drug-dissolved fraction versus time is linear.
First-order	$Q_{t}=Q_{0}\times e^{-k_{1}t}$	(6)	The graphic of the decimal logarithm of the released amount of drug versus time is linear.

where *Q*_0_ is the initial amount of drug; *Q_t_* is the amount of drug remaining at time *t*; *Q_t_/Q_∞_* is the fraction of drug released at time *t*; *k*_0_ and *k*_1_ are the kinetic constants.

To compare the release data of samples containing *P. coronarius*, difference factors were calculated as a model-independent approach [48].

(7) $$f_{1}=\frac{\sum_{j=1}^{n}\left|R_{j}-T_{j}\right|\;\;}{\sum_{j=1}^{n}R_{j}}\times 100$$

where *n* is the sampling number, *R_j_* and *T_j_* are the percent dissolved of the reference and the test products at each time point *j*, respectively.

### 4.11. Biocompatibility Experiments

To evaluate cytotoxicity of the selected excipients and the formulated ointments, MTT assay was performed. The experiments were carried out on HaCaT cell line. HaCaT cells are human immortalized keratinocytes, thus they perfectly represent human skin. The cells were maintained by weekly passages in Dulbecco’s DMEM culture media. For MTT assay, the cells were seeded on a 96-well plate in the density of 10.000 cells/well. When the cells fully grow over the well’s membrane, the experiment is ready to perform. First, we removed the culture media, then we applied the test solutions and incubated the cells with them for 30 min. After 30 min we removed the test substance and added MTT paint solution at 5 mg/mL concentration to the cells (tetrazolium bromide). Then, we let it incubate with the cells for 3 h. The viable cells will transform the water-soluble tetrazolium bromide into formazan precipitate. When the incubation is done, formazan precipitate was dissolved with the isopropanol:hydrochloric acid = 25:1 ratio. Then, the absorbance of these solutions was measured by spectrophotometer (Fluostar Optima) and is directly proportional to the number of viable cells [49,50]. 

### 4.12. Texture-Analysis Experiments

Resistance of the creams was evaluated with the help of CT3 Texture Analyzer (Brookfield, Middleboro, MA, USA). During the investigation, a compression test was performed in normal mode with the following settings: target value (5 mm), target load (4 g), target speed (0.5 mm/s) [43].

### 4.13. Statistical Analysis

Data were analyzed with GraphPad Prism 6 and presented as means ± SD. Comparison of the groups in MTT assays were performed with one-way ANOVA test and *t*-test. Statistical analysis was carried out for antioxidant assays between the leaf and the flower by performing *t*-test. Significant differences on the figures are signed with asterisks. Differences were regarded as significant when *p* ˂ 0.05. All experiments were performed at least in triplicate with the exception of the in vitro time-kill antimicrobial tests where only one experiment was carried out [51]. 

## 5. Conclusions

The present study provides novel and useful information about the chemical and therapeutic properties of a widespread but scientifically less studied plant, *Philadelphus coronarius*. In the experiments, some of the presumed effects of the herb were investigated, utilizing both the flower and the leaf. The identification of chemical compounds and the determination of the exact quantity of the active pharmaceutical ingredients adds to the current knowledge about the plant. Ointments were formulated by incorporating the lyophilized product into the preparations. This raises the possibility of applying the herb topically considering the antimicrobial potential of *Philadelphus coronarius*.

## Figures and Tables

**Table 1:** Bioactive-compound content of *P. coronarius* leaves.

*P. coronarius* Leaf	Bioactive Compound Content (Mg Compound/100 mg) ± SD
7-methoxycoumarin	0.2061 ± 0.032
Chlorogenic acid	0.0128 ± 0.0097
Caffeic acid	0.0740 ± 0.0033
Delphinidin 3-rutinoside chloride	0.3354 ± 0.047
Hyperoside	0.0514 ± 0.0071
Luteolin 7-glucoside	0.2528 ± 0.056
Rutin	0.0941 ± 0.0045
T-resveratrol	0.0602 ± 0.0026

In this table the bioactive-compound content of *P. coronarius* leaf is presented. Data represent the quantity of the isolated components from 100 mg leaf extract. From the components, delphinidin 3-rutinoside chloride, luteolin 7-glucoside and 7-methoxycoumarin are present in a high amount.

**Table 2:** Bioactive-compound content of *P. coronarius* flowers.

*P. coronarius* Flower	Bioactive-Compound Content (Mg Compound/100 mg) ± SD
7-methoxycoumarin	1.6725 ± 0.372
Chlorogenic acid	0.2485 ± 0.098
Ferulic acid	0.1094 ± 0.020
Gallic acid	0.1375 ± 0.034
Rosmarinic acid	0.7674 ± 0.112
Trans p-coumaric acid	0.4387 ± 0.079
Bergapten	2.8370 ± 0.432
Caffeic acid	1.8407 ± 0.087
Delphinidin 3-rutinoside chloride	1.7928 ± 0.201
Diosmin	1.1125 ± 0.386
Hyperoside	0.2428 ± 0.042
Isopimpinellin	0.4678 ± 0.016
Luteolin 7-glucoside	0.0585 ± 0.0093
Myricetin	0.0645 ±0.0021
Quercetin	0.1449 ± 0.034
Rutin	0.4077 ± 0.015
T-resveratrol	0.5262 ± 0.027

In this table the bioactive-compound content of *P. coronarius* flower is presented. It lists the exact quantity of the components isolated from 100 mg flower extract. Bergapten, caffeic acid, delphinidin 3-rutinoside chloride and 7-methoxycoumarin are present in large quantities.

**Table 3:** Antioxidant activity of *P. coronarius* leaf and flower evaluated by different methods.

	*P. coronarius* Leaf	*P. coronarius* Flower
DPPH (%)	86.63 ± 6.49	92.24 ± 10.09
ABTS (mmol TE/g DW)	18.37 ± 4.73	40.54 ± 9.77 *
FRAP (μmol TE/g)	77.97 ± 46.01	164.62 ± 52.75
Cuprac (μmol TE/mL)	222.42 ± 21.04	423.35 ± 71.07 **

In this table antioxidant activity (means ± SD) of *P. coronarius* is presented with different methods. Abbreviations: GAE—Gallic-Acid Equivalent, QE—Quercetin Equivalent, DW—Dry Weight, TE—Trolox Equivalent. * indicates significant difference at *p* ≤ 0.0241; ** indicates significant difference at *p* ≤ 0.0093. Statistical analysis was carried out between the leaf and the flower by performing a *t*-test.

**Table 4:** Total polyphenol and flavonoid content ± SD of *P. coronarius* leaf and flower.

	*P. coronarius* Leaf	*P. coronarius* Flower
Polyphenols (mg GAE/100 g)	59.31 ± 4.36	54.83 ± 9.74
Flavonoids (mg QE/100 g)	1.97 ± 1.49	1.91 ± 0.82

In this table total polyphenol and flavonoid content of *P. coronarius* leaf and flower extract is shown (means ± SD). Abbreviations: GAE—Gallic-Acid Equivalent, QE—Quercetin Equivalent.

**Table 5:** Inhibition of different microbes by *P. coronarius* leaf or flower.

	Inhibition of Microbial Strains by *P. coronarius* Flower or Leaf Measured with Microdilution Method at 5% Concentration ± SD			
	*C. albicans*	*S. aureus*	*E. coli*	*P. aeruginosa*
***P. coronarius* flower**	no inhibition	no inhibition	46.4% ±3.4%	no inhibition
***P. coronarius* leaf**	no inhibition	68.6% ±5.6%	41.5% ±2.7%	no inhibition

In this table cell-viability values of different microbes are presented. *P. coronarius* flower reduced the cell viability of *E. coli*, while the leaf decreased the viability of *S. aureus* and *E. coli*.

**Table 6:** Composition of the six ointment formulations.

	Abbreviation	Leaf or Flower	Emulsifier
Composition 1.	CMP 1	leaf	SP70
Composition 2.	CMP 2	leaf	Tefose 63
Composition 3.	CMP 3	leaf	Sedefos 75
Composition 4.	CMP 4	flower	SP70
Composition 5.	CMP 5	flower	Tefose 63
Composition 6.	CMP 6	flower	Sedefos 75

**Table 7:** Correlation-coefficient values of the compositions.

Kinetic Model		
Composition	Zero	First
CMP 1	0.03	0.37
CMP 2	0.14	0.44
CMP 3	0.59	0.70
CMP 4	0.58	0.71
CMP 5	0.65	0.75
CMP 6	0.392	0.384

**Table 8:** Release-rate and diffusion-coefficient values of the ointments.

Composition	Release Rate (*k*) (μg/cm^2^ × √min)	Diffusion Coefficient (D × 10^−7^; cm^2^/min)
CMP 1	7.47	3.30
CMP 2	8.05	3.97
CMP 3	5.31	2.36
CMP 4	7.45	4.32
CMP 5	6.57	3.97
CMP 6	5.29	1.40

**Table 9:** Difference factors to compare the different release profiles of the ointments.

Composition	*f1*
CMP 1 vs. CMP 2	6.87
CMP 1 vs. CMP 3	31.40
CMP 1 vs. CMP 4	2.98
CMP 1 vs. CMP 5	14.44
CMP 1 vs. CMP 6	24.02
CMP 2 vs. CMP 3	36.11
CMP 2 vs. CMP 4	9.65
CMP 2 vs. CMP 5	20.32
CMP 2 vs. CMP 6	29.24
CMP 3 vs. CMP 4	29.29
CMP 3 vs. CMP 5	24.71
CMP 3 vs. CMP 6	9.71
CMP 4 vs. CMP 5	11.81
CMP 4 vs. CMP 6	21.68
CMP 5 vs. CMP 6	11.19

**Table 10:** Composition (CMP) of the ointments.

	CMP 1	CMP 2	CMP 3	CMP 4	CMP 5	CMP6
Transcutol	+	+	+	+	+	+
SP70 Tefose 63 Sedefos 75	+	−	−	+	−	−
	−	+	−	−	+	−
	−	−	+	−	−	+
*P. coronarius* leaf *P. coronarius* flower	+	+	+	−	−	−
	−	−	−	+	+	+
Cetostearyl alcohol Stearic acid Glycerol IPM	+	+	+	+	+	+
	+	+	+	+	+	+
	+	+	+	+	+	+
	+	+	+	+	+	+
Propylene glycol Purified water	+	+	+	+	+	+
	+	+	+	+	+	+

## Data Availability

Data are available from the corresponding author with the permission of the head of the apartment. The data that support the findings of this study are available from the corresponding author (bacskay.ildiko@pharm.unideb.hu), upon reasonable request.
